# Rare bleeding source in esophageal carcinoma – a case of upper gastrointestinal hemorrhage from intercostal artery pseudoaneurysm: A case report and literature review

**DOI:** 10.1097/MD.0000000000045852

**Published:** 2025-11-14

**Authors:** Liping Ge, Jiannan Shang, Wenqian Tian, Defang Niu, Junshan Hao, Xu Zheng

**Affiliations:** a Department of Interventional Radiography, Heze Municipal Hospital, Heze, Shandong Province, China.

**Keywords:** esophageal carcinoma, intercostal artery, pseudoaneurysm, transcatheter arterial embolization (TAE), upper gastrointestinal hemorrhage (UGIH)

## Abstract

**Rationale:**

Transcatheter arterial embolization (TAE) serves as a critical intervention for upper gastrointestinal bleeding (UGIB) when endoscopic therapy fails or the bleeding source cannot be localized. The intercostal artery is an exceedingly rare source vessel for UGIB. This report describes a case of UGIB caused by a pseudoaneurysm of the left intercostal artery in a patient with esophageal carcinoma, along with a review of the relevant literature.

**Patient Concerns:**

The patient presented to our hospital due to UGIH.

**Diagnoses:**

UGIH.

**Interventions:**

After admission, failure of medical therapy prompted emergency endoscopy, which revealed an active pulsatile bleeding. Following unsuccessful endoscopic hemostasis, emergency TAE was performed, achieving successful embolization of a pseudoaneurysm in the left intercostal artery.

**Outcomes:**

Postoperatively, the patient’s hemoglobin levels remained stable, and no further bleeding was observed.

**Lessons:**

Multidisciplinary collaborative management is essential for UGIB. TAE should be considered when conventional therapies fail. During TAE, if routine angiography of suspected culprit vessels yields negative results, a high index of suspicion for rare ectopic feeding arteries (e.g., intercostal arteries) is required. This case report aims to enhance the diagnostic and embolization proficiency of interventional radiologists in managing UGIB caused by such uncommon aberrant vessels.

## 1. Introduction

UGIH represents a clinically common condition categorized by etiology into variceal and non-variceal bleeding. Acute UGIH most frequently stems from non-variceal sources, particularly peptic ulcer bleeding. Current management primarily involves pharmacotherapy and endoscopic intervention. However, when hemorrhage proves refractory to endoscopic control or when the bleeding source remains unidentified, transcatheter arterial embolization (TAE) serves as the therapeutic alternative. Among culprit vessels, intercostal artery involvement is exceptionally rare. We present a case of UGIH caused by a left intercostal artery pseudoaneurysm in an esophageal cancer patient, accompanied by a review of relevant literature.

## 2. Case profile

A 55-year-old male presented with hematemesis of bright red blood approximately at 2:00 pm on July 12 after eating, accompanied by abdominal distension and nausea. He was initially admitted to a local hospital, where laboratory tests revealed hemoglobin (Hb) of 80 g/L and platelets of 409 × 10⁹/L, with normal coagulation, liver/kidney function, electrolytes, and cardiac enzymes. Despite unspecified therapeutic measures, his condition showed no significant improvement, prompting transfer to our institution for further management. He was admitted to the Gastroenterology Department with a diagnosis of acute UGIH.

Past medical history included surgical resection for esophageal carcinoma. On admission, the patient was conscious but lethargic, with pallor. Cardiopulmonary and abdominal examinations were unremarkable. Repeat blood tests showed a further drop in hemoglobin to 59 g/L. Treatment included pantoprazole for acid suppression, thrombin powder for hemostasis, and somatostatin to reduce splanchnic blood flow, along with transfusion of 2 units of leukocyte-depleted red blood cells. However, the patient experienced another episode of massive hematemesis, with hemoglobin plummeting to 50 g/L, prompting consultation with the critical care medicine team. The consensus recommended emergent bedside endoscopy for definitive diagnosis with potential endoscopic hemostasis. Following transfer to the intensive care unit (ICU), endoscopic evaluation demonstrated:

- Residual esophagus: Preserved peristalsis with congestive and edematous anastomotic mucosa.
- Gastric remnant: Diffuse mucosal congestion/edema, longitudinally oriented folds, extensive adherent blood clots, and active pulsatile bleeding (*No ulcers in esophagus, stomach, or duodenum. Images unavailable due to ICU procedural constraints*).

Our interventional radiology team, having suspected arterial hemorrhage given the pulsatile bleeding pattern, initiated TAE. Sequential angiography of the superior mesenteric artery, esophageal arteries, celiac trunk and its branches (gastroduodenal, left gastric, hepatic, and gastroepiploic arteries) revealed anatomically normal vasculature without contrast extravasation.

**Figure 1.**
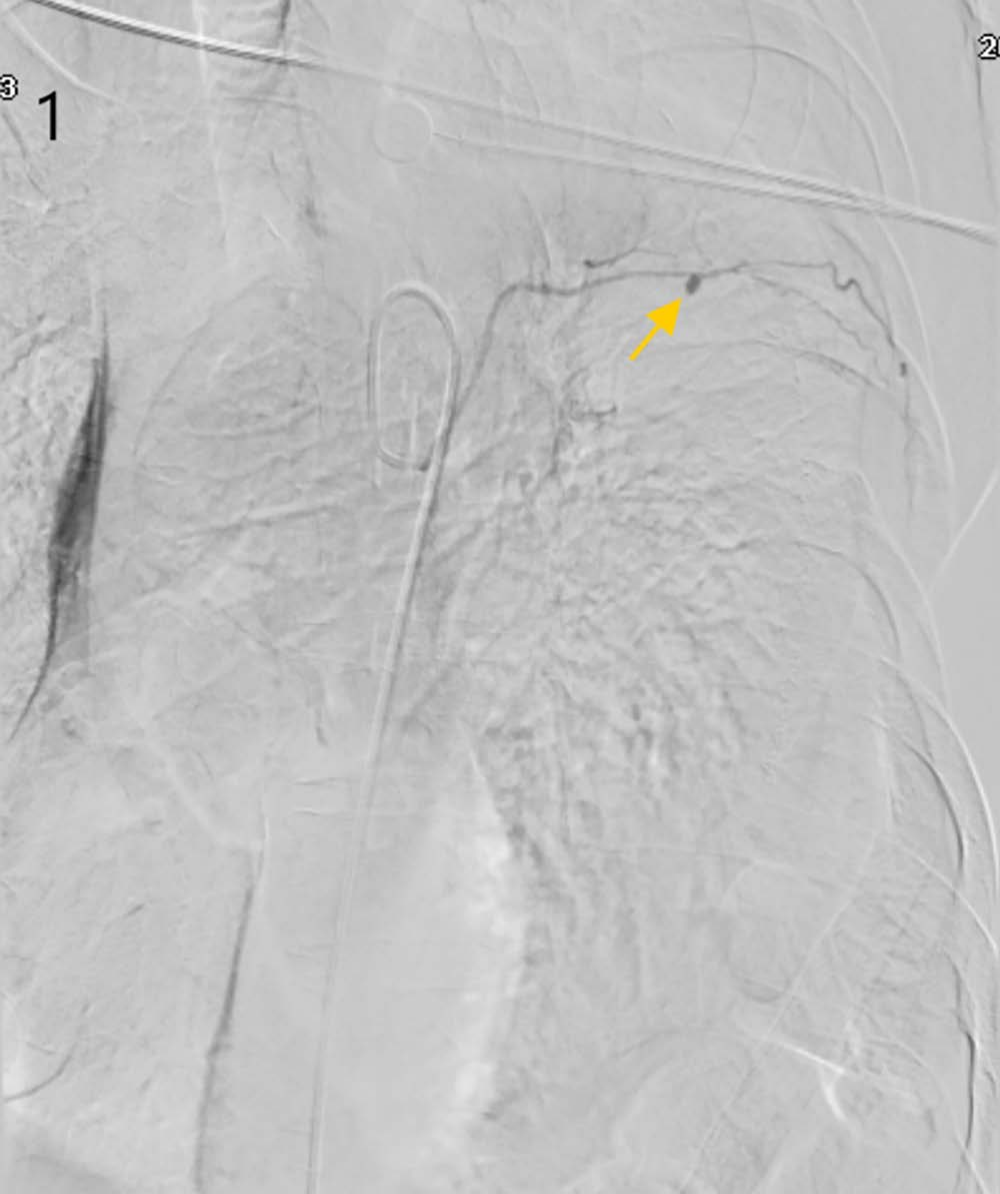
Intraoperative angiography demonstrating the intercostal artery pseudoaneurysm.

**Figure 2.**
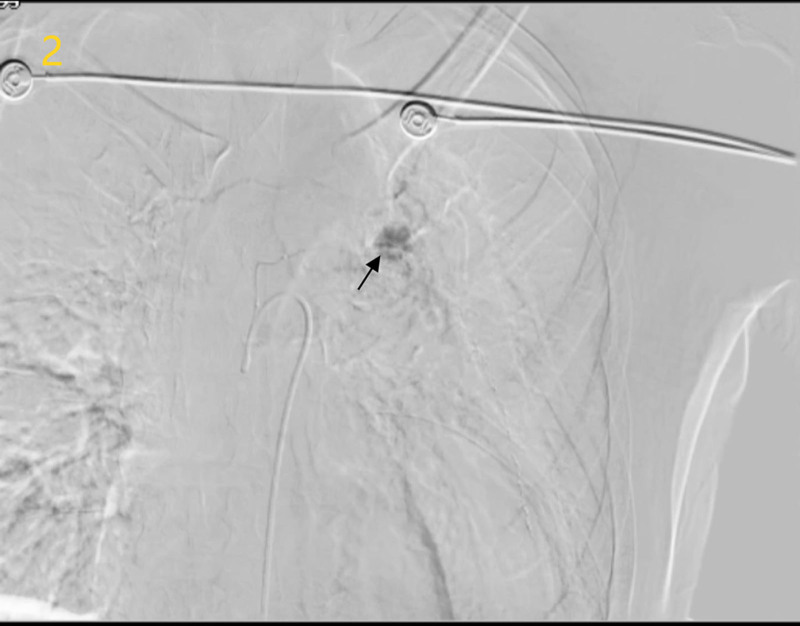
Intraoperative angiographic image (black arrow: contrast extravasation).

Given the absence of bleeding in conventional territories, we hypothesized ectopic vessel involvement – consistent with published post-esophagectomy vascular anomalies where bronchial or intercostal arteries may contribute. Selective catheterization of the left fifth intercostal artery using wire-guided microcatheterization identified a pseudoaneurysm (Fig. 1). Subsequent Intraoperative angiographic image confirmed rupture with active contrast extravasation (Fig. 2, Video S1), definitively localizing the culprit vessel.

**Figure 3.**
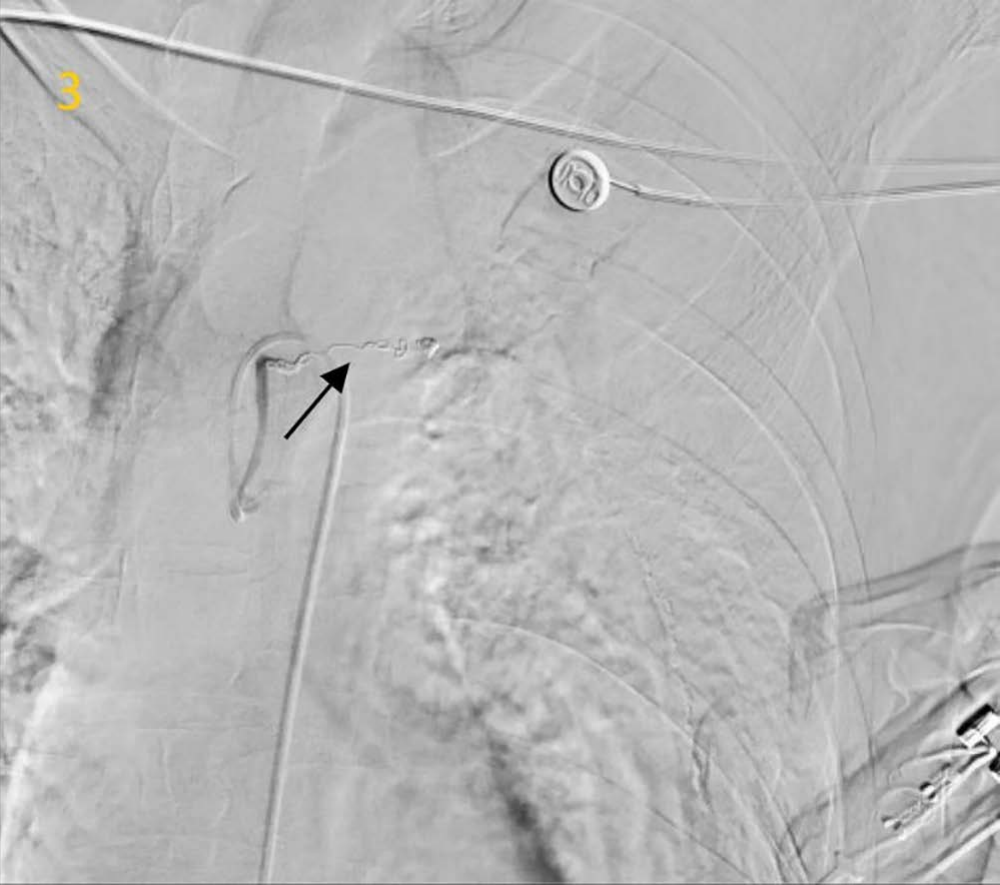
Post-embolization angiography confirming pseudoaneurysm occlusion.

**Figure 4.**
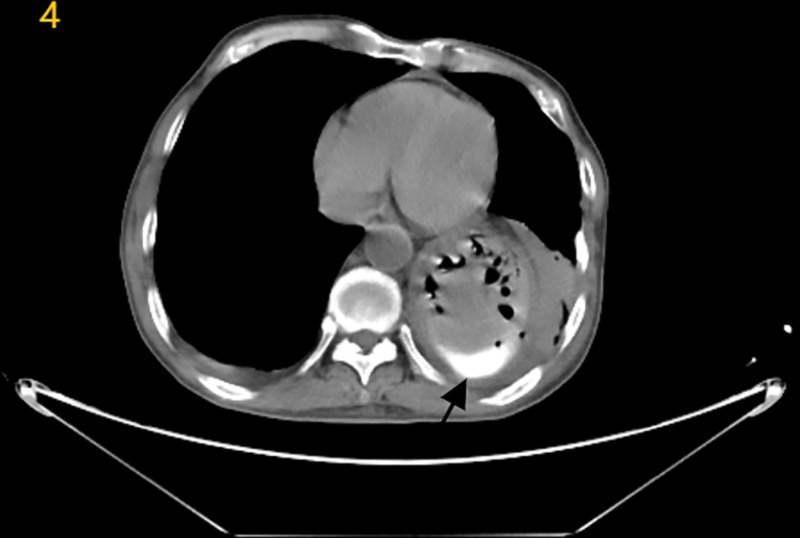
Follow-up CT on postoperative day 2 (arrows: irregular hyperdense material in the gastric lumen, consistent with residual contrast medium from the ruptured pseudoaneurysm).

The lesion was embolized using detachable coils, achieving complete angiographic occlusion (*“cutoff sign,” Fig. 3*). Post-procedural computed tomography (CT) on day 2 demonstrated intragastric hyperdense material (Fig. 4), interpreted as residual contrast medium from the ruptured pseudoaneurysm. The patient remained hemorrhage-free and was discharged to gastroenterology on post-procedure day 3.

## 3. Discussion

UGIH represents a common clinical condition, with pharmacotherapy and endoscopic intervention remaining the mainstay of treatment. However, when endoscopic hemostasis fails or the bleeding source cannot be identified, TAE serves as an effective alternative.^[1]^ Nevertheless, angiographically confirmed UGIH originating from intercostal artery supply is exceptionally rare. In 2006, Kim^[2]^ first reported intercostal artery as the culprit vessel in UGIH, with only sporadic cases documented thereafter. We herein summarize and analyze published cases of intercostal artery-related UGIH (Table 1).

**Table 1:** Patient characteristics and procedure details.

Authors (year)	Gender/age	Presenting symptom	Esophageal cancer	Comorbidity	Surgical/trauma history	CRT	Culprit vessel	Angiographic findings	Embolic materials	Efficacy
Kim et al (2006)	Male, 70 yr	Hematemesis	Yes	Ulcer	Esophagectomy	CT-RT	Rt 5th ICB	CE	GSP, coils	Yes
Lin Z et al (2021)	Male, 71 yr	Hematemesis	Yes	Ulcer	Esophagectomy	CT-RT	Rt 5th ICA	PSA, CE	GSP, coils	Yes
Lin Z et al (2021)	Male, 68 yr	Hematemesis	Yes	Ulcer	Esophagectomy	CT-RT	Rt 8th ICA	PSA	Coils	Yes
Lin Z et al (2021)	Male, 65 yr	Hematochezia/Melena	Yes	Ulcer	Esophagectomy	RT	Rt ICB trunk	CE	Coils, NBCA (1:2)	Yes
Lin Z et al (2021)	Male, 66 yr	Hematemesis	Yes	Ulcer	Esophagectomy	CT-RT	Rt 10th ICA	CE	Coils, NBCA (1:1), GSP	Yes
Taniguchi H et al (2011)	Male, 65 yr	Hematemesis	Yes	Esophago-arterial fistula	None	CT-RT	ICA	NS	NS	Yes
Tajima T et al (2017)	Male, 69 yr	Hematemesis	Yes	Esophago-arterial fistula	None	CT-RT	Rt 7th ICA	PSA	Coils	Yes
Kai C et al (2020)	Male, 43 yr	Hematemesis	No	Esophago-arterial fistula with ulcer	Trauma history	None	Rt 5th ICA	PSA	Coils	Yes
Present case	Male, 55 yr	Hematemesis	Yes	None	Esophagectomy	None	Lt 5th ICA	PSA, CE	Coils	Yes

CE = contrast extravasation, CT-RT = chemoradiotherapy, GSP = gelatin sponge particles, ICA = intercostal artery, ICB = intercostobronchial artery, Lt = left, NBCA = N-butyl cyanoacrylate, PSA = pseudoaneurysm, RT = radiotherapy, Rt = right.

The most frequently implicated arteries in UGIH are the celiac trunk and its branches, along with the superior mesenteric artery. When conventional angiography fails to identify the bleeding source, ectopic feeding vessels should be considered. Notably, in patients with esophageal cancer, esophageal blood supply may involve multiple arteries, including the right intercostal and bronchial arteries, as documented in the literature.^[3]^ Among reported cases, 7 originated from the right intercostal artery, while one did not specify laterality - findings consistent with existing research. Our case demonstrated 2 unique aspects: the left intercostal artery served as the culprit vessel; the left and right intercostal arteries arose independently without a common trunk. These observations underscore the necessity for interventional radiologists to routinely evaluate bilateral intercostal arteries during diagnostic angiography, accounting for potential anatomical variations.

As demonstrated in Table 1, all reported cases of intercostal artery-induced UGIH occurred in male patients. Notably, 7 cases had esophageal carcinoma history, including 5 with prior esophagectomy. Regarding bleeding etiology, postoperative patients primarily exhibited peptic ulcer bleeding, which aligns with the most common causes of UGIH,^[2,4,5]^ while non-operated esophageal cancer patients were presumed to have developed esophago-arterial fistulas. The formation of esophago-arterial fistulas may result from several mechanisms, including direct tumor invasion of vascular structures, radiation- or chemotherapy-induced damage to the vascular wall, and the particular anatomical vulnerability of intercostal arteries in esophageal cancer patients.^[1,6]^ To date, only 3 cases of angiographically confirmed esophago-arterial fistula have been reported in the literature,^[1,7,8]^ with all cases demonstrating intercostal arteries as the responsible bleeding vessels. Importantly, this case represents an uncommon source of postoperative bleeding after esophagectomy for cancer, where upper endoscopy did not reveal any ulceration.

Current literature indicates that chemoradiotherapy may lead to vascular complications, including the formation and rupture of pseudoaneurysms.^[9]^ Additionally, traumatic injury and iatrogenic damage have been implicated in the development of intercostal artery pseudoaneurysms.^[1,10]^ Among the 8 reported cases, 4 demonstrated angiographic evidence of intercostal artery pseudoaneurysms, likely attributable to these etiologies. Notably, the present case is particularly unusual as the patient had no history of trauma, iatrogenic injury, or chemoradiotherapy, yet angiography revealed a spontaneous intercostal artery pseudoaneurysm that was successfully treated with embolization and identified as the culprit source of gastrointestinal bleeding.

Regarding embolization materials, clinical options typically include coils, gelatin sponge, polyvinyl alcohol (PVA) particles, N-butyl cyanoacrylate (NBCA) glue, and Onyx liquid embolic system.^[11]^ Coils, as permanent embolic agents, can effectively occlude target vessels either alone or in combination with other liquid embolic materials.^[12]^ In all reported cases where embolization was performed, coils were exclusively used with favorable follow-up outcomes, supporting their safety and efficacy for this indication.

Therapeutic outcomes analysis demonstrates that all patients who underwent successful identification and embolization of the culprit vessel achieved positive results, confirming TAE as a safe and effective treatment for refractory UGIH. In our case, post-embolization hemoglobin levels stabilized without recurrent bleeding, indicating treatment success. However, the patient unfortunately succumbed to tumor progression 4 months after discharge.

## 4. Conclusion

In the management of UGIB, multidisciplinary team (MDT) collaboration stands as the cornerstone, with the close coordination among gastroenterologists, radiologists, and interventional radiologists being particularly pivotal. TAE serves as a crucial salvage therapy when conventional treatments fail, and its efficacy is highly contingent upon the precise identification of the bleeding focus and any potential ectopic feeding vessels. Preprocedural abdominal contrast-enhanced CT can accurately localize the bleeding site and delineate ectopic vasculature, thereby providing critical guidance for TAE. In this case, in the absence of such preoperative CT guidance, the interventional team successfully identified and embolized an ectopic intercostal artery supply, achieving effective hemostasis through their extensive experience, meticulous angiographic exploration, and heightened vigilance for ectopic blood supply. This underscores the significant practical value of interventionalists’ experience and vigilance in the clinical management of UGIB, particularly when searching for sources of ectopic arterial supply.

**Special note:** Due to the emergent nature of the patient’s condition, bedside endoscopy was performed in the ICU setting, and consequently, no endoscopic images were obtained for documentation.

## Acknowledgments

The authors would like to express their gratitude to Qingyong Fan for the expert linguistic services.

## Author contributions

**Formal analysis:** Defang Niu, Junshan Hao, Xu Zheng.

**Investigation:** Jiannan Shang.

**Methodology:** Xu Zheng.

**Resources:** Wenqian Tian.

**Software:** Junshan Hao.

**Writing – original draft:** Liping Ge.
